# Large to massive rotator cuff tendon tears: a protocol for a systematic review investigating the effectiveness of exercise therapy on pain, disability and quality of life

**DOI:** 10.12688/hrbopenres.13242.1

**Published:** 2021-07-15

**Authors:** Kathryn Fahy, Rose Galvin, Jeremy Lewis, Karen McCreesh

**Affiliations:** 1 School of Allied Health, University of Limerick, Castletroy, Munster, V94T9PX, Ireland; 2 Ageing Research Centre, Health Research Institute, University of Limerick, Limerick, Ireland; 3 School of Health and Social Work, University of Hertfordshire, Hatfiield, Hertfordshire, AL10 9AB, UK; 4 Therapy Department, Central London Community Healthcare, National Health Service Trust, London, UK; 5 Department of Physical Therapy & Rehabilitation Science, Qatar University, Doha, Qatar

**Keywords:** Shoulder, Rotator Cuff Tendon Tear, Exercise, Physiotherapy, Rehabilitation, Conservative Management/ Intervention, Non-surgical management/ Intervention

## Abstract

**Background: **Rotator cuff tendon tears are inextricably linked with the natural process of aging often resulting in severe disability, poor quality of life and an added burden to the health care system. The occurrence of rotator cuff tendon tears increases exponentially with every decade of life to approximately 60% in individuals over 80 years of age. Exercise is a commonly prescribed intervention although research on its efficacy is in its infancy and often conflicting. The purpose of this systematic review is to investigate the effectiveness of exercise interventions for people diagnosed with large to massive rotator cuff tendon tears.

**Methods: **This systematic review will adhere to the PRISMA reporting guidelines. A comprehensive search of five databases will be conducted. Randomised clinical trials (RCT) or quasi-randomised control trials will be included if they evaluate exercise as the core intervention or as part of the intervention in the management of large to massive rotator cuff tears. To quantify response to treatment we will compare changes in pain, disability and quality of life (QoL). The Consensus on Exercise Reporting Template (CERT) will be used to characterise the different types of exercise intervention. The Cochrane Risk of Bias Tool will be used to assess study quality.

A narrative synthesis with meta-analysis will be performed, and the certainty of evidence will be assessed using the Grading of Recommendations Assessment, Development and Evaluation (GRADE) criteria.

**Discussion: **This review will synthesise the totality of GRADE A and B evidence on the effectiveness of exercise for large to massive rotator cuff tendon tears. It will provide clinically important information and guidance for immediate implementation by clinicians, health policymakers and may be used to guide future research.

**PROSPERO registration: **244502 (24/03/2021)

## Introduction

The rotator cuff muscles originate from the scapula and fuse to form a tendon that encompasses the humeral head to provide both stability and movement of the glenohumeral joint. Rotator cuff (RC) tendon tears are the most commonly observed shoulder pathology in the adult population, with a prevalence rate of 30–60% in people over the age of 60 years of age ( Tempelhof *et al.*, 1999; Teunis *et al.*, 2014). While many tears remain asymptomatic, two in every three people with a large to massive tear (2 or more tendons, > 5cm) will develop symptoms that include but are not limited to, recurrent and persistent pain, a painful arc of movement in abduction, weakness in shoulder abduction and/or external rotation and night pain ( Edwards *et al.*, 2016). The shoulder symptoms are commonly associated with sleep disturbances and an inability to perform many of the physical activities valued by the individual ( Moosmayer *et al.*, 2013). The prognosis is highly uncertain with 40–50% of people reporting persistent pain and disability 6–12 months after onset ( Kuijpers *et al.*, 2006). When left untreated these large to massive rotator cuff tears may result in cuff tear arthropathy ( Eljabu *et al.*, 2015) for which the main treatment option is a reverse shoulder arthroplasty (RSA). This involves removing the ends of the two bones and fitting a prosthetic shoulder joint.

For most people undergoing surgery the risks of post-surgical complications of deep infection, bony fracture and instability of the prosthesis are commonly balanced with a substantial reduction in pain and improvement in quality of life ( Cowling *et al.*, 2017). Unfortunately, the post-operative course is not as certain for others, who report ongoing or worsening pain and no improvement in function, and some failure of the prosthesis. This usually results in more complex surgery and is associated with substantial economic cost for the health care system and often the individual ( Jain & Yamaguchi, 2014). Unsuccessful surgery leads to ongoing physical limitations, depression, anxiety and loss of independence that many perceive to have the same subjective psychological impact as other persistent medical conditions such as heart failure ( Martinez-Calderon *et al.*, 2018). With an aging population and an odds ratio increase of 2.69 of a RC tear for every decade of life (p = 0.005) ( Duong *et al.*, 2020) combined with the associated disability, establishing treatments that align with patients goals is of high priority for researchers, clinicians, health economists and society.

Despite the high prevalence of RC tears, best management approaches for people experiencing symptomatic large to massive RC tears remains uncertain. Historically the trend in management has favoured a surgical intervention with rotator cuff repairs almost tripling in the United Kingdom over the 14 years to 2009 ( Ensor *et al.*, 2013). In the following decade, there has been a substantial growth in the number of randomised control trials (RCTs), cohort studies and systematic reviews of shoulder treatments ( Agout *et al.*, 2018; Boorman *et al.*, 2018; Christensen *et al.*, 2016). The most recent Cochrane review concluded that exercise offers equivalent outcomes to surgery for mild or moderate rotator cuff injury but the evidence remains uncertain in its transferability to full thickness tears especially involving the subscapularis ( Karjalainen *et al.*, 2019).

A systematic review using data up to 2016 which examined exercise in the management of large rotator cuff tears indicated moderate strength evidence for the benefits of exercise, but it also highlighted the lack of well-designed clinical trials at this time. In addition the exercise intervention was not quantified by type, duration or frequency ( Jeanfavre *et al.*, 2018). Two recent systematic reviews focusing on conservative management of massive irreparable rotator cuff tears ( Kovacevic *et al.*, 2020; Shepet *et al.*, 2020) again highlighted the lack of high quality comparative studies to help inform treatment recommendations. Shepet *et al.* (2020) utilised efficacious non operative treatment to collate and provide a synthesised rehabilitation program for this population cohort to help guide the treating clinicians, something which has never been completed for large rotator cuff tears.

A randomised clinical trial comparing a progressive exercise programme to usual care in full thickness tears indicated promising outcomes in shoulder pain and function ( Ainsworth *et al.*, 2009). Again, the efficacy of exercises has been demonstrated but the optimal dosage remains unknown. This has been echoed in a number of cohort studies that have concluded that exercise can lead to significant improvements in shoulder pain and function and reduce the necessity for surgery, in people with large tears of their rotator cuff tendons ( Boorman *et al.*, 2014; Kuhn *et al.*, 2013). The heterogeneity of exercise programmes across all studies has provided difficulty in synthesising the data and establishing robust evidence-based rehabilitation programmes. We know that consistent components of the rehabilitation programmes include strengthening, range of motion, stretching/flexibility, activity modification/education, home exercise routine, postural interventions, heat/cold modalities and manual therapy ( Jeanfavre *et al.*, 2018). Strengthening is most prescribed and is the main component of exercise rehabilitation for this condition.

With no gold standard exercise programme for large to massive rotator cuff tears, often interventions are based on established rehabilitation programmes for other conditions ( Kuhn, 2009). Despite being commonly prescribed the evidence to support exercise in the non-surgical management of large to massive rotator cuff tendon tears remains equivocal.

The objectives of this review are to:

(1) Synthesise the evidence on the effectiveness of exercise interventions compared to other interventions or a control, in improving clinical and functional outcomes (shoulder pain, function and quality of life) in adults with large to massive rotator cuff tendon tears of the glenohumeral (shoulder) joint. 

(2) Determine the optimal exercise intervention using the CERT checklist in terms of the specific parameters (type, dose, intensity, frequency) of the intervention to improve outcomes in adults with large to massive rotator cuff tendon tears.

## Methods

This protocol has been developed according to the preferred reporting items for systematic reviews and meta-analyses guidelines for systematic review protocols (PRISMA-P) ( Fahy, 2021; Moher *et al.*, 2015). The study is registered on PROSPERO (244502, 24 ^th^ March 2021).

### Research question

*What is the effectiveness of exercise therapy on pain, disability and quality of life in people with large to massive rotator cuff tendon tears?*

### Eligibility criteria

The following criteria will be used to select studies for inclusion in the systematic review:

***Study design.*** Randomised control trials (RCTs) and quasi-randomised control trials will be included. Cohort studies, case reports, case control studies, editorials, letters, viewpoints and studies that are published in abstract only will be excluded.

***Setting.*** All healthcare settings (hospital, community, health centre) and all geographical locations will be included.

***Population.*** Adults (18 years of age or older) with large to massive rotator cuff tendon tears which meet one or more of the following criteria; two or more tendons, size of the tear being at least 3 cm or non-operable, confirmed with magnetic resonance imaging (MRI), ultrasound or arthrography. It will also include patients with concomitant shoulder conditions such as osteoarthritis secondary to rotator cuff tear arthropathy (RCTA) confirmed by radiographic examination. Participants will be excluded if they have experienced traumatic tendon tears or fractures, experienced neurological signs, adhesive capsulitis (frozen shoulder), shoulder instability and systemic inflammatory diseases such as rheumatoid arthritis. In studies that have a mix of aetiology, we will include the study where over 80% of the population meet the inclusion criteria outlined above on the aetiology of rotator cuff tear.

***Interventions.*** Exercise is defined as “a series of specific movements with the aim of training or developing the body by a routine practice or as physical training to promote good physical health” ( Abenhaim *et al.*, 2000). We will include studies examining the effectiveness of any type of shoulder exercise intervention (active supported, closed chain, active mobilisation with resistance, cuff rehabilitation or perturbations) as a standalone intervention or as part of an active exercise multimodal approach (strengthening, range of motion, flexibility). Interventions that combine exercise with passive or alternative modalities such as joint mobilisation, Injection therapy (corticosteroids), pain – relieving medication or any form of analgesia will also be included, only if it was offered to patients in both trial groups.

***Comparators.*** The comparators of interest will be non-surgical interventions (passive, exercise or usual care) or surgical interventions.

***Outcomes.*** Any standardised assessment of self-reported pain and disability (combined) and/or health related quality of life.

***Additional outcome(s).*** Range of motion, strength and surgical intervention within one year.

***Language.*** Only English language studies will be included, however the number of non-English language papers identified will be recorded.

### Information sources

The databases to be searched from inception to April 2020: EBSCO (Medline and CINHAL), PubMed, Cochrane Library and PEDro. The search for unpublished studies will include ClinicalTrials.gov and Cochrane Central Register of Controlled Trials. Only full texts available in the English language are to be included due to a lack of translation resources.

Limits imposed on the search: human and older than 18 years of age.

### Search strategy

**Figure 1.**
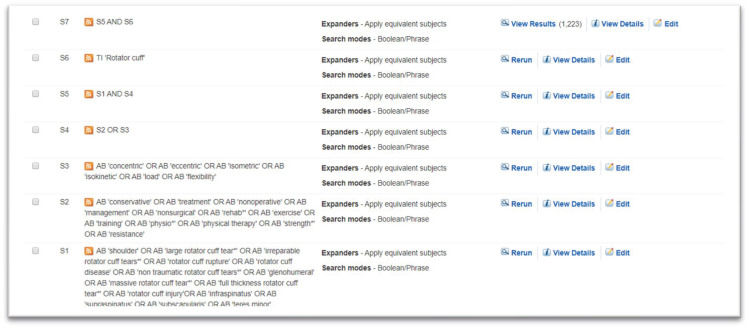
Sample search strategy – Cinahl on the EBESCO Platform.

The search strategy was developed by the primary author (KF) in collaboration with a Health Science Librarian (LD) with an expertise in systematic review searching. Keywords were derived from the research question along with reviewing recent literature on the topic, with input from all authors ( Table 1). LD was consulted on formulating an initial search for Medline (EBSCO Platform) as well as translating the search to other databases and utilising the respective MeSH terms. The search will be rerun and updated before the final analysis is conducted. The search terms and a sample search strategy are shown in Table 1 and Figure 1, respectively.

**Table 1.** Search terms (keywords/ MeSH* terms).

Rotator Cuff	Shoulder, Glenohumeral, Irreparable rotator cuff tears, Rotator cuff rupture*, Full thickness rotator cuff tear, Massive rotator cuff tear*, Large rotator cuff tear, Rotator cuff disease, Rotator cuff injury, Non traumatic tears, Infraspinatus, Supraspinatus, Teres Minor, Subscapularis.
Exercise	Conservative management, Treatment, Non-operative management, Non-surgical, Rehab*, Exercise*, Training, Physiotherapy, Physical therapy, Strength*, Concentric, Eccentric, Isometric, Isokinetic, Resis*, Load, Flexibility

### Study records

***Data management.*** Literature search results will be exported to EndNote X9 and duplicate records selected using the ‘remove duplicates’ function and by manually screening results of accuracy (KF). Search results will then be imported to Rayyan QCRI, a web-based software platform designed to support abstract screening and collaboration among multiple authors. A second search for duplicate records will be performed in Rayyan QCRI.

***Selection process.*** One researcher (KF) will initially screen all identified studies by title and then title and abstract and finally by full text. Articles clearly not meeting the established inclusion/exclusion criteria will be excluded. Two independent researchers (KF and KMcC) will then be involved in screening the article title and abstract identified for full text review and screen the full texts for inclusion. If there is disagreement about inclusion, the abstract will be reviewed by a third author (JL) to determine suitability.

The reference lists of included studies will be scanned to identify any relevant additional studies that may have been omitted. Additionally, the reference lists of relevant systematic reviews will be cross checked to ensure all applicable publications are identified. Both reviews will independently screen the full text of these additional articles to determine inclusion/exclusion (KF and KMcC).

A PRISMA flow diagram will outline the overall process of study selection and give details on inclusion and exclusion of studies at each stage. If necessary, study authors will be contacted to resolve any eligibility queries (KF). All reasons for excluding articles will be reported.

***Data collection process.*** A standardised data extraction tool ( Table 2) will be developed specifically for this review based on recommendations provided in the Cochrane Handbook of Systematic Reviews of Interventions ( Higgins & Green, 2011)(KF). Using the standardised form, that will have been piloted to ensure accuracy, two review authors (KF and KMcC) will independently extract the following pertinent study characteristics from included studies:

**Table 2.** Data extraction table (Cochrane Handbook of Systematic Reviews of Interventions) ( Higgins & Green, 2011).

Author Year	Participants	Tear type & Diagnostic Criteria	Intervention Groups Length of Intervention	Clinical outcome scores (time points)	Results
1.					
2.

1. *Participants:* Number of participants allocated to each treatment group, gender, and mean age/age range, tear size/type, diagnostic criteria, intervention groups and length of intervention.
2. *Outcomes:* Primary and secondary clinical outcomes scores will be specified and collected at identified time points, effect size, between group difference and results mean.

A second data extraction tool ( Table 3) using the 16 item CERT ( Slade *et al.*, 2016) will be used to characterise the different types of exercise interventions that have been evaluated in the included studies:

3. *Interventions/exposure:* Type/duration and frequency of exercise, supervised or unsupervised, group or individual, how adherence was measured, inclusion of individual exercises/ progressions or generic programmes.

**Table 3.** Exercise intervention data extraction table: Consensus on Exercise Reporting Template (CERT) ( Slade *et al.*, 2016).

Section/Topic	Item #	Check List Item	Description of Study X	Location (Page, table, appendix)
WHAT: materials	1	Detailed description of the type of exercise equipment (e.g weights, exercise equipment such ergometer etc)		
WHO: Provider	2	Detailed description of the qualifications, teaching/supervising expertise, and/or training undertaken by the exercise instructor		
HOW: Delivery	3	Describe whether exercises are performed individually or in a group		
	4	Describe whether exercises are supervised or unsupervised and how they are delivered		
	5	Detailed description of how adherence to exercise is measured and reported		
	6	Detailed description of motivation strategies		
	7a	Detailed description of the decision rule(s) fir determining exercise progression		
	7b	Detailed description of how the exercise program was progressed		
	8	Detailed description of each exercise to enable replication (e.g. photographs, illustrations, video etc)		
	9	Detailed description of any home program component (e.g. other exercises, stretching etc)		
	10	Describe whether there are any non-exercise components (e.g. education, cognitive behavioural therapy, massage etc)		
	11	Describe the type and number of adverse events that occurred during exercise		
WHERE: location	12	Describe the setting in which the exercises are performed		
WHEN, HOW MUCH: dosage	13	Detailed description of the exercise intervention including, but not limited to, number of exercise repetitions/sets/sessions, session duration, intervention/program duration etc		
TAILORING: what, how	14a	Describe whether the exercises are generic (one size fits all) or tailored whether tailored to the individual.		
	14b	Detailed description of how exercises are tailored to the individual.		
	15	Describe the decision rule for determining the starting level at which people commence an exercise program ( such as beginner, intermediate, advances etc)		
HOW WELL: planned, actual	16a	Describe how adherence or fidelity to the exercise intervention is assessed/ measured.		
	16b	Describe the extent to which the intervention was delivered as planned		

If necessary, study authors will be contacted up to three times to provide further details (KF). A third researcher (JL), will be contacted if there are any disagreements or differences in opinion about data extraction. One review author (KF) will transfer data into the RevMan file. 

### Outcomes and prioritisation

The primary outcomes of interest will be self-reported pain and disability (individual or combined) and health related quality of life. Secondary outcomes will be range of motion, strength and surgical intervention within one year. Outcomes collected at baseline, 3 months, 6 months and yearly will be included in the review.

### Risk of bias

Methodological quality of the included RCTs will be rated using the Cochrane risk of Bias tool ( RoB 2.0) ( Higgins *et al.*, 2011). The risk of Bias tool covers five domains and assesses how trial conduct may bias results resulting in more or less reliable evidence ( Eldridge *et al.*, 2016). Two independent reviewers (KF and RG) will review the study quality. Should any discrepancies arise a third reviewer (JL) will be contacted. Where data is missing all attempts will be made to contact the primary authors for clarification.

### Data synthesis

The Cochrane Review Manager software ( RevMan 5) will be used to conduct all statistical analyses. As a measure of exercise impact, the mean difference (MD) with 95% confidence interval (CI) between the exercise and the control group will be used as the mode of analysis. In studies where the median is reported, the median will be used as a proxy of the mean and a multiple of 0.75 times the interquartile range will be used as a proxy for the standard deviation ( Hozo *et al.*, 2005). In studies where different outcomes are used to measure the same construct (e.g. pain), a standardised mean difference (SMD) will be reported with 95% CI.

Heterogeneity across the studies will be evaluated using the I ^2 ^statistic, which calibrates the amount of variation, by cause of heterogeneity rather than chance. For values of approximately 25%, 50% and 75%, the extent of inconsistency in the studies’ results will be considered low, moderate and high ( Higgins *et al.*, 2003). I ^2 ^greater than 50% will be considered as substantial heterogeneity. If I ^2^ is less than or equal to 50% a fixed affect meta-analysis will be used. Where I ^2^ is greater than 50%, a random effects model will be applied.

***Analysis of subgroups or subsets.*** Sensitivity or subgroup analyses will be conducted to explore the individual study characteristics (such as age, tear size or location or comparator) in order to identify potential sources of heterogeneity (clinical and methodological variation). 

### Meta-bias(es)

Publication bias will be examined in the studies by visually inspecting the funnel plots generated in the meta-analysis.

### Confidence in cumulative evidence

The Grading of Recommendations Assessment, Development and Evaluation ( GRADE) working group criteria will be used to evaluate the current level evidence of exercise therapy (as an individual intervention or as part of a multimodal non-operative intervention) and provide a grade of recommendation. The GRADE working group has developed a hierarchal, alphabetical letter scale of A to F ( Table 4) which considers the quality of evidence and strength of recommendations to facilitate applying research directly to clinical decisions and inform health care policy.

**Table 4.** Grading of Recommendations Assessment, Development and Evaluation (GRADE) working group criteria. ( Schunemann, 2008).

Grade of Recommendation		Strength of Evidence
A	Strong	A preponderance of level I and/or level II studies support the recommendation. Must include ≥ 1 level I study.
B	Moderate	A single high-quality randomized controlled trial or a preponderance of level II studies support the recommendation.
C	Weak	A single level II study or a preponderance of level III and level IV studies including statements of consensus by content experts support the recommendation.
D	Conflicting	Higher-quality studies conducted on this topic disagree with respect to their conclusions. The recommendation is based on these conflicting studies.
E	Theoretical/ Foundational	A preponderance of evidence from animal or cadaver studies, from conceptual models/ principles, or from basic sciences/bench research support this conclusion.
F	Expert Opinion	Best practice based on the clinical experience of the guidelines development team.

### Ethical approval and consent to participate

Ethical approval is not required for this study as it will not involve or include personal data or conduct experimental research with humans.

## Discussion

Exercise is commonly prescribed in the treatment of large to massive rotator cuff tears despite the conflicting evidence on its effectiveness. While the body of evidence investigating its effectiveness has grown significantly, there is need to pool the evidence to accurately measure treatment effect and the factors that may contribute to some of the reported benefits.

This will be the first systematic review of exercise in the effectiveness in the treatment of large to massive rotator cuff tears that will incorporate the individual characteristics of the exercise intervention into the analysis using randomised control trials only. Thus far there is limited evidence identifying the optimal mode, frequency, intensity and duration of exercise that should be recommended for maximum benefit. By exploring these factors, the findings from this review may assist clinicians and surgeons in deciding to recommend or prescribe exercise as a first choice intervention for large to massive rotator cuff tears.

## Dissemination of information

The findings of the systematic review will be published in a peer-reviewed journal upon completion. This systematic review will be of interest not only to researchers and academics but also healthcare professionals working in this field and thus the findings will be presented at the Irish Society of Chartered Physiotherapists national annual conference. Key findings will be disseminated via social media platforms of the research team, e.g. Twitter.

## Study status

The search strategy has been completed and piloted in relevant databases.

## Data availability

No data are associated with this article.

### Reporting guidelines

PRISMA-P checklist for ‘Large to massive rotator cuff tendon tears: a protocol for a systematic review investigating the effectiveness of exercise therapy on pain, disability and quality of life’. http://doi.org/10.5281/zenodo.4680984 ( Fahy, 2021).

Data are available under the terms of the Creative Commons Attribution 4.0 International license (CC-BY 4.0).
