# Supplementary material for: Large to massive rotator cuff tendon tears: a protocol for a systematic review investigating the effectiveness of exercise therapy on pain, disability and quality of life
Source: HRB Open Res. 2021 Sep 30;4:75. Originally published 2021 Jul 15. [Version 2] doi: 10.12688/hrbopenres.13242.2 (PMC8385455; doi:10.12688/hrbopenres.13242.2)
Supplement: Peer review history

# Peer review: Reviewer response for version 1

**DOI:** 10.21956/hrbopenres.14405.r29863

**Published:** 2021-08-24

**Authors:** Ingrid Hultenheim Klintberg

**Affiliations:** 1 Sahlgrenska Academy, Institute of Neuroscience and Physiology, Department of Health and Rehabilitation, Unit of Physiotherapy, University of Gothenburg, Gothenburg, Sweden; 2 Sahlgrenska University Hospital, Mölndal, Sweden

Comments to the study protocol:

- The protocol is thoroughly planned and the research process well described in all its steps. 
- The objectives and rationale for the planned systematic review are comprehensively and thoroughly described. 
- I have no criticism of the research methodology as here described. However, as I do not have expertise in statistics, I cannot comment on the description of the planned statistics. 
- For clarity, state that the study will assess non traumatic early in the *Introduction* section. 
- The Introduction may also benefit from a patho-anatomic description of the development of non traumatic rotator cuff tears. As the rationale for the exercise selection might be linked to this process and how to reduce its influence on pain and dysfunction or even prevent the propagation of the tear. However, this may also be more thoroughly described in the final paper when you present your results of the review. 
- The challenge to withdraw results to inform clinicians from separate studies evaluating exercise therapy is clearly presented thus the clinical value of the planned study is clearly presented. 

Nevertheless, I have a few queries. 

- Under *Methods, Population:*
  "Participants will be excluded if they have experienced traumatic tendon tears"
  What are your cut off lines for a traumatic tendon tear? It will be difficult to validate if authors responsible for patient selection to separate studies and you are in agreement. 
  This is difficult and I understand that you must have measures to handle it in a pragmatic way. It would strengthen the credibility of the paper if this difficulty would be addressed. 
- Another description within physiotherapy that is difficult is "Usual care". When used in your selected studies, I would recommend you to get more details. The word "usual" gives the impression of low quality.
- There are two typing errors. Page 5, left column and third paragraph: ... Both reviewers (instead of *reviews*).
- Table 3, point 7a: ...decision rule(s) for determining... (instead of *fir*)

A few additional thoughts, these are not criticism, just thoughts that have arised while scrutinizing your plan:

-The CERT extraction table is very helpful, however, I miss the description of assessment of quality in exercise performance. The patient adhere perfectly, but have not the skills to adjust performance according to the purpose of the exercise. Will the exercise be beneficiary or provocative?
- Have you discussed how to group different types of exercises? Do you group them by muscle group or by the rationale of its effect?

The physiotherapy community both researchers and clinicians will have a lot to learn from the results and discussions of your planned review

Is the study design appropriate for the research question?

Yes

Is the rationale for, and objectives of, the study clearly described?

Yes

Are sufficient details of the methods provided to allow replication by others?

Yes

Are the datasets clearly presented in a useable and accessible format?

Not applicable

Reviewer Expertise:

PhD in the field of rotator cuff dysfunction. Clinically active and treat patients with rotator cuff dysfunctions or other shoulder trauma on a daily basis. My last publication is a systematic review on cryotherapy.

I confirm that I have read this submission and believe that I have an appropriate level of expertise to confirm that it is of an acceptable scientific standard.

# Peer review: Reviewer response for version 1

**DOI:** 10.21956/hrbopenres.14405.r29862

**Published:** 2021-07-28

**Authors:** Ian Horsley

**Affiliations:** 1 English Institute of Sport, Manchester, UK

This study aims to synthesise the evidence on the effectiveness of active exercise interventions in comparison to other interventions or a control, on improving clinical and functional outcomes of patients who have been diagnosed as having large to massive rotator cuff tears at the shoulder.

 The authors have an excellent research profile and are highly regarded internationally within the field of management of shoulder dysfunctions.

 The proposal is detailed and should  provide valuable information regarding optimal management of this condition and will guide further research in this area

Is the study design appropriate for the research question?

Yes

Is the rationale for, and objectives of, the study clearly described?

Yes

Are sufficient details of the methods provided to allow replication by others?

Yes

Are the datasets clearly presented in a useable and accessible format?

Yes

Reviewer Expertise:

Rehabilitation of the shoulder, especially in sport

I confirm that I have read this submission and believe that I have an appropriate level of expertise to confirm that it is of an acceptable scientific standard.
